# One patient, one destiny: A cluster analysis of the Parkinson’s progression Markers Initiative (PPMI) cohort

**DOI:** 10.1016/j.prdoa.2026.100437

**Published:** 2026-03-21

**Authors:** Khashayar Dashtipour, Mehrbod Vakhshoori, Zahra Hashempour, Alberto J. Espay, Mark Ghamsary, Jacob Jones, Farzin Pedouim, Karen Frei, Ava Baghaei

**Affiliations:** a Department of Neurology, Loma Linda University Health System, Loma Linda, CA, USA; b Internal Medicine Department, AdventHealth Orlando, Orlando, FL, USA; c School of Medicine, Shiraz University of Medical Sciences, Shiraz, Iran; d James J. and Joan A. Gardner Family Center for Parkinson’s Disease and Movement Disorders, Department of Neurology, University of Cincinnati, Cincinnati, OH, USA; e School of Public Health, Loma Linda University, Loma Linda, CA, USA; f Department of Psychology, Center On Aging, California State University San Bernardino, San Bernardino, CA, USA

**Keywords:** Parkinson’sdisease, Heterogeneity, PPMI, Genetic, Individualizedtreatment

## Abstract

**Background:**

Parkinson’s disease (PD) is characterized by heterogeneity. While previous efforts have aimed to categorize patients by clinical subtypes, these classifications have not reliably predicted outcomes, underscoring the complexity and variability of disease progression.

**Methods:**

Using data from the Parkinson’s Progression Markers Initiative (PPMI), we conducted a longitudinal analysis of 209 individuals with PD over five years. We used cluster analysis to identify trajectory clusters in the progression of motor symptoms. Participants were categorized based on baseline variables, including age, sex, race, BMI, genetic mutation status, medical history, presence of jaw and limb tremors, and alpha-synuclein seeding status. Motor and non-motor outcomes, such as gait abnormalities, dyskinesia, motor fluctuations, dystonia, cognitive impairment, hallucinations, and others, were evaluated, along with the progression of the levodopa equivalent daily dose (LEDD). Sankey plots illustrated patient trajectories from baseline to outcome groups over time.

**Results:**

Trajectory cluster analysis of MDS-UPDRS Part III scores identified three progression patterns: slow, intermediate, and rapid, with higher baseline BMI associated with faster motor progression (P < 0.01). Grouping based on baseline features identified 136 distinct patient groups, with substantial dispersion across clinical outcomes. Patients with identical baseline profiles frequently diverged into separate outcome groups. Genetic/SAA stratification also showed marked variability in progression with no significant association across trajectory clusters (P > 0.05).

**Conclusion:**

These findings highlight the existence of highly individualized clinical trajectories, even among patients with similar baseline characteristics, within the specific set of baseline variables examined in this study. This emphasizes the need for a personalized, patient-centered approach in both the management and study of Parkinson’s disease.

## Highlights

• Patients with identical baseline features showed divergent 5-year outcomes.
• Genetic and α-synuclein status did not predict clinical trajectories.
• Findings underscore the need for individualized, patient-centered PD care.

## 1 Introduction

The variability of Parkinson’s disease (PD) symptoms is both profound and remarkable [1]. Motor manifestations occur in diverse phenotypes, with patients exhibiting different degrees of tremor, rigidity, bradykinesia, and gait disturbances. The progression of these symptoms varies widely across individuals. Non-motor symptoms frequently coexist with motor dysfunction, further underscoring the heterogeneity of the disease [2]. However, not all non-motor symptoms are present in every patient, and their severity and distribution differ significantly. Comorbid conditions, such as cardiovascular, endocrine, and other neurodegenerative disorders, add further complexity to clinical presentations. Regardless of the initial symptom profile, disease progression remains highly variable and often unpredictable. Each patient appears to follow a distinct disease course, likely shaped by a combination of genetic factors, environmental exposures, lifestyle, and pathophysiological mechanisms.

This heterogeneity is also evident at the pathological level [3], [4]. Autopsy studies of PD patients reveal varied patterns of protein accumulation [3]. Notably, Lewy bodies (LBs) may be absent in some cases [5]. Mixed pathologies, including multiple protein aggregates alongside vascular changes, are increasingly recognized and serve as further indicators of diverse underlying mechanisms [6]. This pathological variability is observed not only in sporadic PD but also in monogenic forms of the disease [7].

Genetic studies reinforce this complexity. Genome-wide association studies (GWAS) have identified over 90 genetic variants associated with PD risk, and the number continues to grow as analyses include more diverse populations [8]. Clinical heterogeneity is likewise reflected in therapeutic response: patients enrolled in clinical trials often exhibit markedly different responses to the same investigational treatments [9].

Given the extensive variability in genetic makeup, pathogenesis, environmental exposures, and lifestyle factors, we propose that each patient represents a unique and highly complex disease state. Therefore, attempts to rigidly profile or subtype all PD patients may be overly reductive.

To explore this hypothesis, we applied cluster analysis to the Parkinson’s Progression Markers Initiative (PPMI) dataset. Previous longitudinal studies have identified distinct progression subtypes in PD [10], [11], [12]. However, to date, no study has definitively addressed whether patients with similar baseline characteristics follow parallel disease trajectories or diverge in ways that are independent of comorbidities, genetic variation, or demographic influences. This study aims to answer that question.

## 2 Materials and Methods

This study utilized data from the PPMI, a multicenter observational cohort. The detailed methodology of PPMI has been previously described [13]. Data were finalized in December 2024. Eligible participants were PD patients with ≥ 5 years of available follow-up, and annual assessments per PPMI protocol. We performed trajectory cluster analysis of MDS-UPDRS Part III scores from baseline to year 5 to identify motor progression patterns. MDS-UPDRS Part III assessments were performed in the clinically defined OFF state at baseline and annual visits, as specified in the PPMI protocol.

Baseline (input) variables: age group (<50, 50–65, 65–80, >80 years), sex, race/ethnicity, body mass index (BMI) category (<18.5, 18.5–25, 25–30, >30 kg/m^2^), genetic mutation status (LRRK2, GBA, SNCA, PRKN, or PINK1 vs negative), comorbidity (cardiovascular, endocrine, both, or none), tremor (yes/no), and alpha-synuclein seeding amplification assay (SAA) (positive/negative).

Five-year outcomes (outputs): hallucinations, freezing of gait, speech impairment, difficulty arising from a chair, gait abnormality, postural stability, posture, dyskinesia, motor fluctuations, dystonia, cognitive impairment, depression, anxiety, apathy, dopamine dysregulation syndrome, lightheadedness, fatigue, and levodopa equivalent daily dose (LEDD) increase (positive/negative), all coded as binary or ordinal variables.

Outcome groups were constructed using a combinatorial profiling approach, in which each patient’s outcome profile was defined by the combination of values across the included variables, and groups were formed by exact matching on that full set. Using this approach, we combined all 18 binary and ordinal outcome variables at the 5-year timepoint; two participants were assigned to the same outcome group only if they matched on every included outcome. For ordinal variables (e.g., gait abnormality: normal/mild/severe), we retained the ordered categories consistent with standard clinical interpretation of the MDS-UPDRS response structure. Baseline input groups were also characterized from the predefined baseline variables, with participants assigned to the same input group only if they matched on every included variable. Baseline BMI categories followed World Health Organization cutoffs, and age was grouped into pragmatic categories for profile construction; both were modeled as continuous variables in regression analyses. Subjects with missing data for specific input/outcome variables were excluded from grouping analyses involving those variables. To limit unnecessary data loss when grouping patients, missing comorbidity category values were replaced with the most common category in the cohort. This complete-case approach ensured that grouping and trajectory analyses were based on a consistent set of observed variables; no formal comparisons were made between included and excluded participants.

### 2.1 Statistical analysis

Categorical variables were summarized as counts (percentages), and continuous variables as mean ± SD and median [IQRs]. Model-based functional clustering identified groups with similar MDS-UPDRS Part III longitudinal trajectories using penalized thin-plate splines (R package *clustra*), with spline flexibility constrained (maximum degree of freedom = 10) to avoid overfitting. We performed no single-value imputation for missed visits at intermediate timepoints, and trajectories were estimated using spline smoothing across all available observations per participant. The optimal cluster number was chosen using the lowest Akaike Information Criterion (AIC). We evaluated clustering stability using multiple random initializations and consistently obtained the same 3 clusters across runs.

Group differences were tested with the Kruskal-Wallis test and Dunn’s post hoc test for continuous variables, and Chi-square or Fisher’s exact test for categorical variables, with Bonferroni correction for multiple comparisons. Linear regression identified baseline predictors of progression. Linear regression models examined associations between baseline variables and progression. The multivariable model included age (continuous), sex, race/ethnicity, BMI (continuous), genetic mutation status (positive/negative), comorbidity category (cardiovascular, endocrine, both, or none), baseline tremor status (yes/no), and SAA status as covariates.

Patients were also stratified by combined baseline and outcome profiles to define subgroups with shared characteristics. Sankey plots illustrated transitions between baseline and five-year states. Additional analyses examined progression by combined genetic/SAA status (per SynNeurGe staging), using Fisher’s Exact Test. All analyses were performed in RStudio (v4.4.2; RStudio Inc., Boston, MA). P < 0.05 was considered statistically significant.

## 3 Results

### 3.1 Baseline Characteristics of the Study Population

A total of 209 patients met inclusion criteria. Mean baseline age was 60 ± 9 years; 55% were aged 50–65. Genetic variants were identified in 33% of patients. Comorbidities included cardiovascular disease in 27%, endocrine disorders in 6.7%, both in 23%, and none in 43%. Tremor at baseline was present in 38%. SAA was positive in 91% of tested individuals **(Supplementary Table 1)**.

### 3.2 Five-Year follow-up outcomes

Hallucinations and freezing of gait occurred in 13% and 11% of patients, respectively. Gait abnormalities were noted in 21% (17% mild, 3% severe), and postural deformities in 26% (19% mild, 7% severe). Dyskinesia and motor fluctuations developed in 33% and 48%, while dystonia occurred in 14%. Cognitive impairment emerged in 40%, depression in 27%, anxiety in 43%, and apathy in 23%. Dopamine dysregulation was reported in 9%. Autonomic symptoms were frequent; lightheadedness in 37% and fatigue in 65%. LEDD increased in 93% of participants over five years **(Supplementary Table 2)**.

### 3.3 Trajectory cluster analysis

**Fig. 1:**
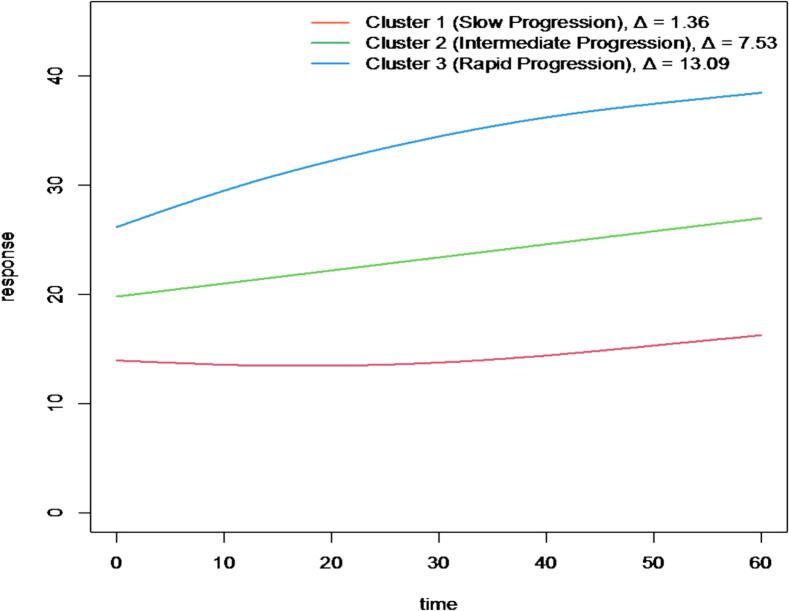
Trajectory cluster analysis of Parkinson’s disease progression based on MDS-UPDRS Part III over time (Cluster 1: N = 73, Cluster 2: N = 77, and Cluster 3: N = 59).

Functional clustering of MDS-UPDRS Part III trajectories identified three progression patterns: slow (Δ = 1.36), intermediate (Δ = 7.53), and rapid (Δ = 13.09) (AIC = 90.89; BIC = 143.43). Median [IQR] baseline and Year-5 MDS-UPDRS Part III scores were 14 [9–17] and 14 [8–17] for the slow cluster (n = 73), 17 [15–21] and 25 [21–32] for the intermediate cluster (n = 77), and 25 [19–30.5] and 34 [28.5–42] for the rapid cluster (Fig. 1).

Among baseline variables, only BMI differed significantly across clusters (P < 0.001). Higher BMI was associated with faster motor progression in both univariate and multivariate models (β = 0.59–0.70, P < 0.01), after adjusting for age, sex, race/ethnicity, genetic status, comorbidities, and tremor status (**Supplementary Table 3**). Full regression results are presented in **Supplementary Table 5**.

### 3.4 Grouping and dispersion analysis

**Fig. 2.1:**
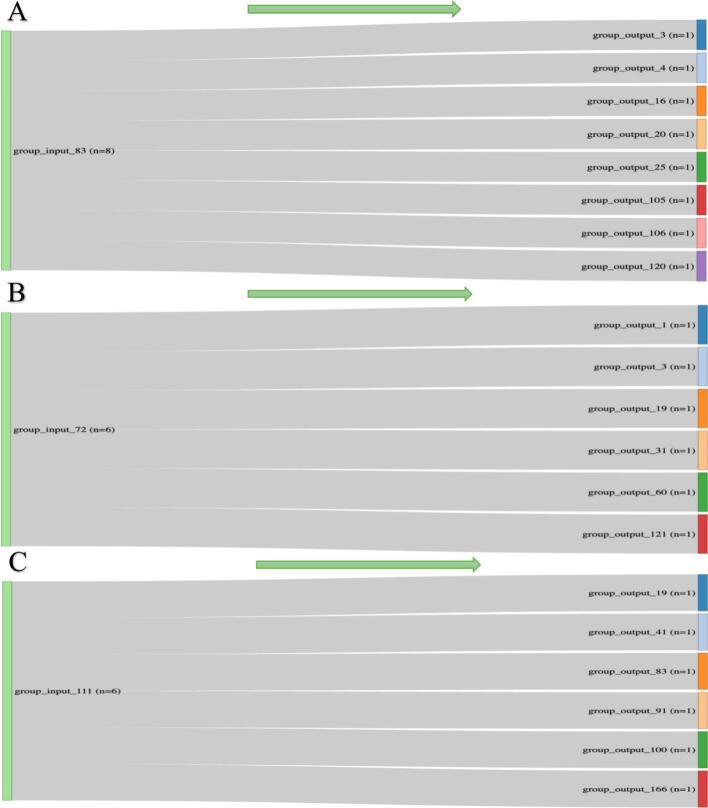
. Sankey plot illustrating the flow of patients within same input groups. A: males aged 50–65 years, White, with negative genetic status, a history of CVD and endocrine, negative tremor, positive SAA status, and a BMI 25–30 kg/m^2^ (N = 8), B: males aged 50–65 years, White, with negative genetic status and comorbidities, negative tremor, positive SAA status, and a BMI 25–30 kg/m^2^ (N = 6), C: males aged 65–80 years, White, with negative genetic status, positive CVD and endocrine disorders, negative tremor, positive SAA status, and a BMI 25–30 kg/m^2^ (N = 6), Arrows indicate the direction of patient flow.

**Fig. 2.2:**
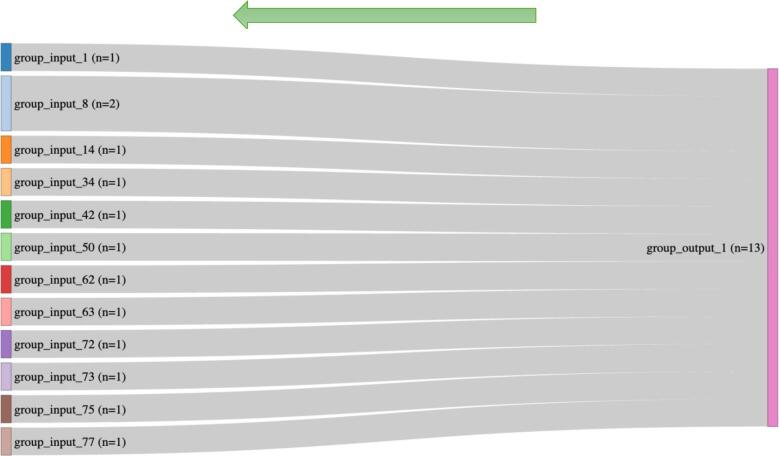
. Sankey plot illustrating the flow of patients within same output group (negative / normal for hallucination, freezing of gait, speech impairment, arising from chair, gait abnormality, postural stability, posture, dyskinesia, fluctuation, dystonia, cognitive impairment, depression, anxiety, apathy, dopamine dysregulation, lightheadedness, fatigue, positive for levodopa equivalent daily dose (LEDD) progression) (N = 13), Arrows indicate the direction of patient flow.

Stratification by baseline characteristics yielded 136 unique subgroups (1–8 patients each), which dispersed into multiple distinct outcome groups, illustrating marked clinical heterogeneity **(**Fig. 2.1, Fig. 2.2**).** Most baseline groups of meaningful size contributed to multiple distinct outcome groups, underscoring that baseline similarity does not guarantee longitudinal similarity in the studied variables. Further stratification by genetic and SAA status defined four categories:

1. Genetic–/SAA– (n = 2),
2. Genetic–/SAA+ (n = 137),
3. Genetic+/SAA– (n = 16),
4. Genetic+/SAA+ (n = 54).

These corresponded to 2, 109, 16, and 52 outcome groups, respectively. No significant association was found between genetic/SAA.

categories and MDS-UPDRS Part III trajectory clusters (Fisher’s exact P = 0.358); however, this finding should be interpreted with caution given the small sample sizes in the genetic−/SAA− (n = 2) and genetic+/SAA− (n = 16) categories **(Supplementary Fig. 1) (Supplementary Table 4)**.

## 4 Discussion

The concept of “one patient, one destiny” underscores a clinical reality familiar to neurologists managing PD. Patients with similar baseline profiles often follow divergent trajectories. This variability, evident in both clinical practice and trials, underscores the challenge of predicting individual responses to treatment. Longitudinal cohorts, such as the PPMI, aim to capture this complexity by collecting extensive biospecimen data and clinical assessments over time, ultimately striving to identify demographic, genetic, and biomarker profiles that enable precision medicine [13]. Previous longitudinal studies in PPMI and other cohorts have successfully identified clinical predictors of progression and distinct disease subtypes, highlighting the value of comprehensive baseline characterization [10], [11], [12].

In this study, longitudinal cluster analysis of MDS-UPDRS Part III trajectories over five years in 209 PD patients identified three distinct progression patterns: slow, intermediate, and rapid. Except for body mass index (BMI), baseline demographic and clinical variables did not differ significantly among clusters. Linear regression, adjusting for age, sex, race/ethnicity, genetic status, comorbidities, and tremor status, identified BMI as independently associated with faster motor progression.

These findings suggest that the specific set of baseline demographic and clinical variables examined in this study has limited predictive value for individual patient outcomes in PD. The previous studies about the effect of the BMI on the progression of PD were inconclusive, with some analyses contradicting others [14]. Some studies indicated a poor outcome on motor and nonmotor aspects of PD with lower BMI, and on the other hand, some studies show a worse outcome of PD symptoms in overweight patients or those with obesity [15]. The observed BMI–progression association (approximately 0.6–0.7 MDS-UPDRS Part III points per kg/m2 over five years) represents a statistical finding within our predefined multivariable model and likely results in only modest differences in motor scores at the individual level. This finding should be viewed as hypothesis-generating and needs replication before being considered a clinically actionable predictor of motor progression. To further elucidate this complexity, we grouped PD patients from a cross-sectional cohort according to similar input and outcome variables. Our findings highlight significant clinical heterogeneity even when only a limited set of variables are considered, underscoring the challenges in accurately predicting long-term outcomes. It is important to note that our analysis was limited to a predefined set of baseline variables and did not incorporate some of the PD-specific measures, including disease duration, age at symptom onset, baseline MDS-UPDRS Part III score, baseline Hoehn and Yahr stage, and baseline levodopa equivalent daily dose (LEDD). These variables are known predictors of progression in PD and their omission represents a key limitation of our grouping schema. Notably, the two largest input groups, consisting of eight and six subjects respectively, exhibited identical predefined baseline characteristics yet diverged significantly over a five-year follow-up, resulting in distinctly different clinical trajectories. The robustness of this observation was illustrated by a reverse Sankey plot, tracing outcomes back to initial inputs. Furthermore, repeating the analysis using the SynNeurGe staging produced comparable results: the two largest groups, 137 subjects (genetically negative, SAA positive) and 54 subjects (genetically positive, SAA positive), diverged into 109 and 52 unique trajectories, respectively. These findings underscore a critical research gap, emphasizing the need for a deeper understanding of PD's long-term variability beyond conventional phenotypic classifications.

Our reliance on Sankey plots to illustrate outcome heterogeneity provides valuable descriptive insights but does not constitute formal predictive modeling. While we report quantitative measures of dispersion, we did not perform comprehensive predictive analyses with discrimination metrics. Such analyses, though beyond the scope of the present Short Communication, represent an important future direction and could more rigorously quantify the predictive value of various baseline profiling approaches. Furthermore, our outcome definitions, primarily binary or coarse ordinal (presence/absence of symptoms, mild/moderate/severe categories), likely oversimplify the underlying spectrum of disease heterogeneity. Also, our choice to categorize continuous variables (age, BMI) into groups, while facilitating the formation of discrete input strata for visualization, may have reduced our ability to detect non-linear relationships between baseline characteristics and outcomes. Future studies should leverage validated continuous severity scales and multi-domain outcome measures (e.g., detailed motor scores, cognitive assessments, quality of life indices) to capture more nuanced progression patterns.

Several factors may explain the limited success of profiling in predicting individual patient outcomes. Below, we discuss these contributing factors and the implications derived from this perspective.

### 4.1 Complexity of Parkinson’s disease

Emerging evidence increasingly supports the characterization of PD as a syndrome rather than a single disease entity [1]. Pathological studies consistently reveal variability in protein aggregates, even among patients sharing similar clinical phenotypes or monogenic forms of PD [16]. Aggregates involving α-synuclein, tau, amyloid-β, TDP-43, and microvascular pathology have all been documented [16]. Genetic studies similarly highlight PD's pathogenic complexity, with notable variability in clinical presentations and disease progression among individuals carrying identical monogenic mutations [4]. Gene ontology analyses further illustrate that implicated genetic variants involve diverse biological pathways, including mitochondrial dysfunction, vesicular transport anomalies, impaired proteostasis, and immune dysregulation [17], [18]. This inherent pathological and genetic heterogeneity significantly complicates predictive modeling based on patient profiles alone.

This genetic and pathogenic heterogeneity manifests not only at the phenotypic and pathological levels but also extends to electrophysiological features, further contributing to the complexity of PD. While early electrophysiological studies identified elevated beta-band activity in the subthalamic nucleus (STN) as a correlate of motor symptoms, particularly bradykinesia, subsequent analyses have revealed some variability in these recordings [19]. Not all patients exhibit exaggerated beta oscillations, and the relationship between beta power and symptom severity is not universally consistent. This individual variability in beta-band activity, especially in recordings from the STN, represents yet another layer of heterogeneity, this time at the electrophysiological level, underscoring the multifaceted nature of PD and the ongoing challenges in fully understanding its underlying mechanisms [19].

### 4.2 The combinatorial challenge

Similar to other chronic and neurodegenerative disorders, combinatorial analytics of clinical and biological variables further contributes to the unpredictability of patient outcomes [20]. Historically, the etiopathogenesis of PD has been oversimplified, often viewed through linear models that attempt to identify responsible genes or pathways (e.g., LRRK2, PINK1, SNCA) or isolate environmental factors (e.g., pesticide exposure, nicotine use), and propose straightforward therapeutic interventions (e.g., exercise). Today, we recognize that PD results from complex interactions involving potentially hundreds or thousands of genes, each contributing small effects rather than the large effects observed in monogenic Mendelian rare diseases [8]. Beyond genetics, additional influences, including epigenetic, transcriptomic, epidemiological, environmental factors, comorbidities, and interactions with medications, shape each patient’s clinical manifestation and trajectory.

Each patient represents a unique combination of genetic predispositions, sex, age, race, BMI, comorbid conditions, medications, epigenetic modifications, lifestyle choices, environmental exposures, and more. Viewed combinatorially, the number of potential patient profiles surpasses the actual population of PD patients, suggesting that each patient’s profile is inherently unique from disease onset, even before divergent clinical trajectories become apparent.

### 4.3 Implications for clinical practice and research

This profound heterogeneity carries significant implications for clinical care and research. It highlights the importance of adopting a patient-centered approach and cautions against overgeneralizing prognosis or treatment response. Furthermore, recognizing the complexity of PD provides valuable insights into the limited success observed with disease-modifying therapies to date. Acknowledging this complexity should motivate a shift toward individualized therapeutic goals that are achievable and meaningful for each patient, alongside sustained investment in fundamental research aimed at elucidating underlying disease mechanisms.

Ultimately, although large-scale cohort studies and multi-omic profiling efforts promise substantial advancements in our understanding of PD, the present analysis suggests that even with extensive baseline characterization using the variables available in this study, individual trajectories remain highly variable. Whether more comprehensive profiling approaches, incorporating detailed imaging, multi-omic data, or longer follow-up periods, can improve predictive accuracy remains an important question for future research.

Several additional limitations warrant consideration. First, our study focused on patients with at least 5 years of follow-up, and progression patterns may require longer observation periods to fully stabilize. Second, the non-significant association between genetic/SAA status and trajectory clusters may reflect insufficient statistical power due to small sample sizes in certain categories (particularly genetic−/SAA − with n = 2). Third, our analysis does not account for treatment effects or medication changes over the follow-up period, which may influence observed trajectories. Finally, our analysis, based solely on MDS-UPDRS Part III trajectories, captures only the motor domain and is influenced by dopaminergic treatment effects. Incorporating multiple clinical domains (e.g., Hoehn and Yahr stage, cognitive function via MoCA, motor complications) would provide a more comprehensive and potentially more predictive characterization of disease progression. Our findings are therefore only applicable to participants with sufficient data for longitudinal profiling, and future research should formally investigate differences between complete and incomplete cases.

Collectively, our findings reinforce the intrinsic variability and complexity of PD trajectories. They emphasize the urgent need for innovative research methodologies capable of unraveling individualized disease progression pathways, ultimately enhancing predictive accuracy and enabling personalized therapeutic strategies, albeit likely achievable only in selected cases.

## CRediT authorship contribution statement

**Khashayar Dashtipour:** Writing – review & editing, Writing – original draft, Visualization, Validation, Supervision, Software, Resources, Project administration, Methodology, Investigation, Formal analysis, Data curation, Conceptualization. **Mehrbod Vakhshoori:** Writing – review & editing, Writing – original draft, Visualization, Validation, Software, Resources, Project administration, Methodology, Investigation, Formal analysis, Data curation, Conceptualization. **Zahra Hashempour:** Writing – review & editing, Writing – original draft, Visualization, Validation, Software, Resources, Project administration, Methodology, Investigation, Formal analysis, Data curation, Conceptualization. **Alberto J. Espay:** Writing – review & editing, Writing – original draft, Validation, Data curation, Conceptualization. **Mark Ghamsary:** Writing – review & editing, Writing – original draft, Validation, Formal analysis, Data curation, Conceptualization. **Jacob Jones:** Writing – review & editing, Writing – original draft, Validation, Data curation, Conceptualization. **Farzin Pedouim:** Writing – review & editing, Writing – original draft, Validation, Data curation, Conceptualization. **Karen Frei:** Writing – review & editing, Writing – original draft, Validation, Data curation, Conceptualization. **Ava Baghaei:** Writing – review & editing, Writing – original draft, Validation, Investigation, Data curation, Conceptualization.

## Declaration of competing interest

The authors declare that they have no known competing financial interests or personal relationships that could have appeared to influence the work reported in this paper.
