# Supplementary material for: One patient, one destiny: A cluster analysis of the Parkinson’s progression Markers Initiative (PPMI) cohort
Source: Clin Park Relat Disord. 2026 Mar 21;14:100437. doi: 10.1016/j.prdoa.2026.100437 (PMC13049996; doi:10.1016/j.prdoa.2026.100437)
Supplement: Supplementary Data 1 [file mmc1.docx]

**Supplementary Figure 1. Sankey plot illustrating the flow of patients within the same input groups. A: negative genetic status, negative SAA (N=2), B: negative genetic status, positive SAA (N=137), C: positive genetic status, negative SAA (N=16), D: positive genetic status, positive SAA (N=54). Arrows indicate the direction of patient flow.**


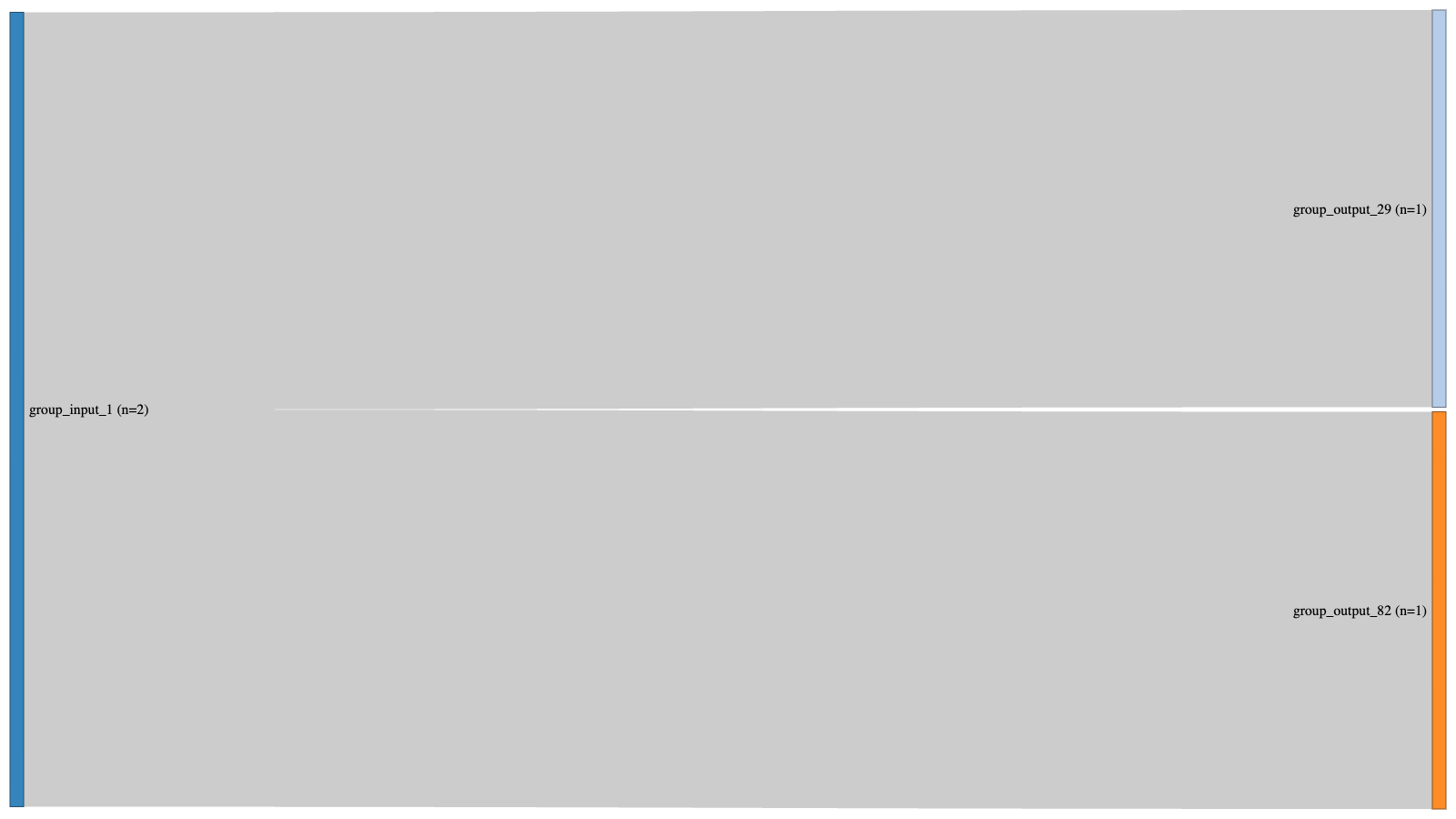

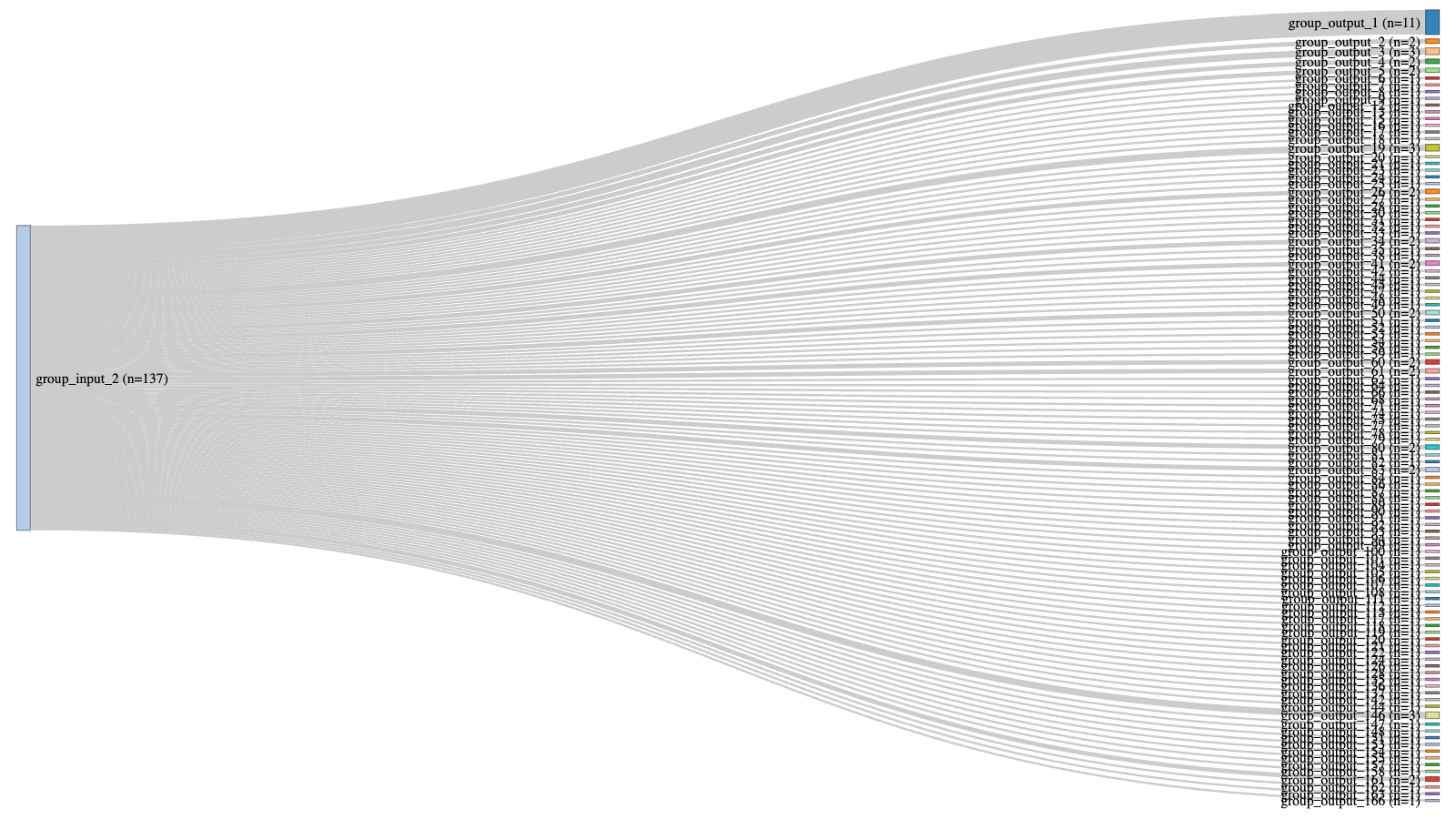

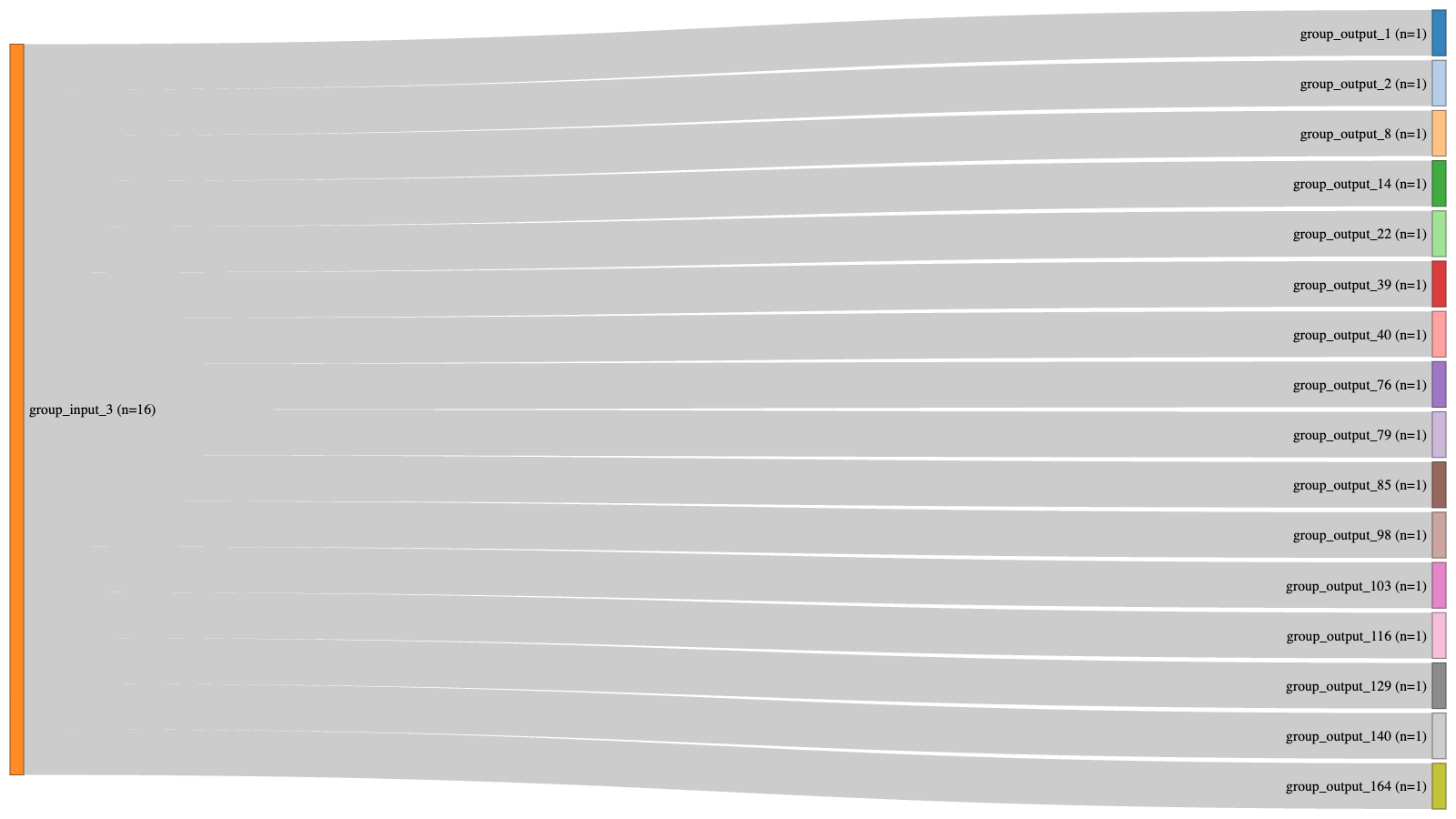

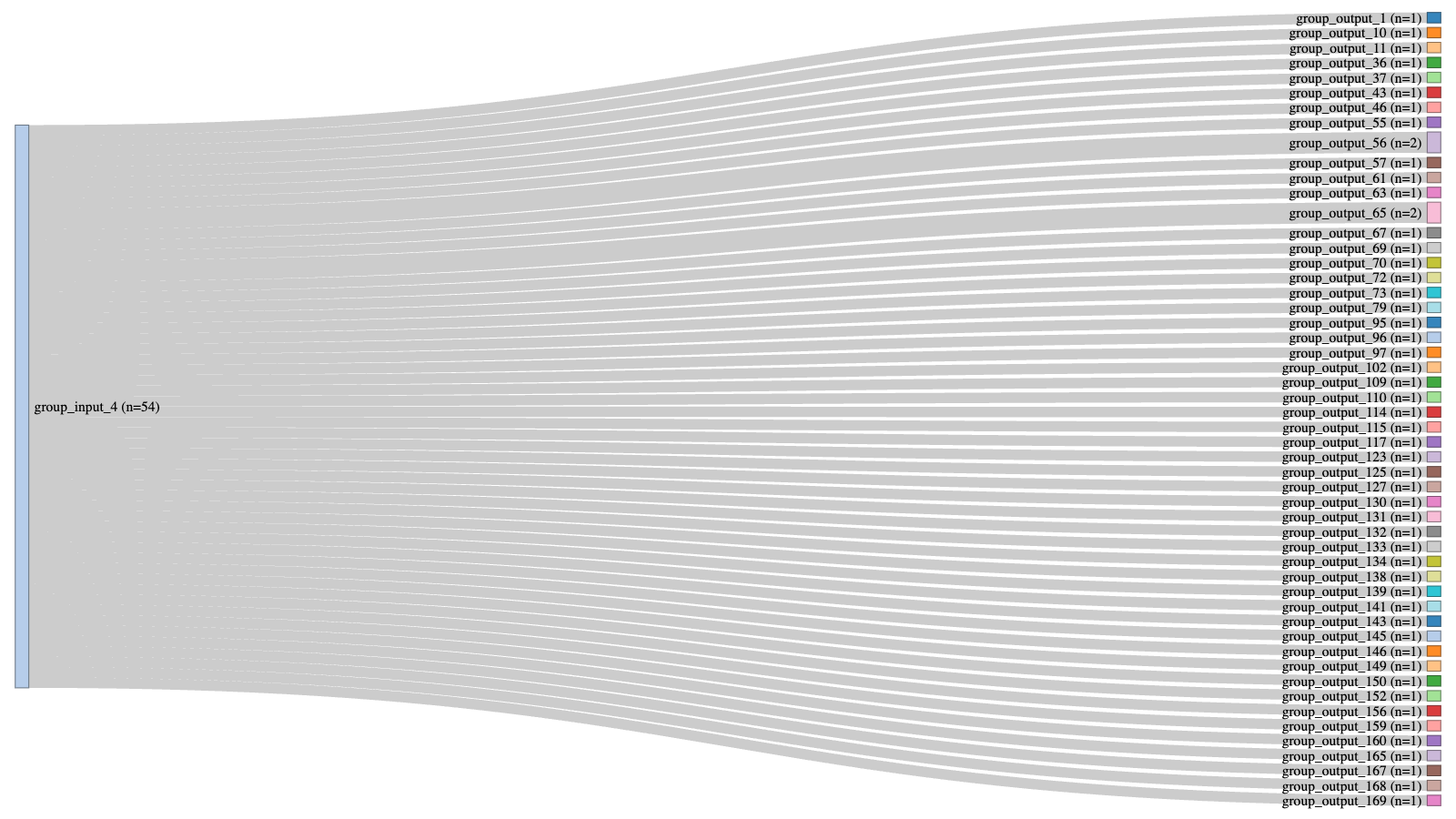


A

B

C

D
