# Supplementary material for: One patient, one destiny: A cluster analysis of the Parkinson’s progression Markers Initiative (PPMI) cohort
Source: Clin Park Relat Disord. 2026 Mar 21;14:100437. doi: 10.1016/j.prdoa.2026.100437 (PMC13049996; doi:10.1016/j.prdoa.2026.100437)
Supplement: Supplementary Data 2 [file mmc2.docx]

**Supplementary Table 1. General characteristics of study population (Input variables)**

| Variables | | N = 209 |
| --- | --- | --- |
| Age (years) | | 60.7 [53.9–66.8] |
| Age category | <50 years | 29 (14%) |
|  | 50-65 years | 115 (55%) |
|  | 65-80 years | 64 (31%) |
|  | >80 years | 1 (0.5%) |
| Sex | Female | 78 (37%) |
|  | Male | 131 (63%) |
| Race | White | 183 (88%) |
|  | Black | 4 (1.9%) |
|  | Asian | 18 (8.6%) |
|  | Others | 4 (1.9%) |
| BMI (kg/m^2^) | | 26.22 [23.81–29.09] |
| BMI category | <18.5 kg/m^2^ | 1 (0.5%) |
|  | 18.5-25 kg/m^2^ | 80 (38%) |
|  | 25-30 kg/m^2^ | 89 (43%) |
|  | >30 kg/m^2^ | 39 (19%) |
| Genetic status | Negative | 139 (67%) |
|  | Positive | 70 (33%) |
| Comorbidities | Negative | 90 (43%) |
|  | Positive cardiovascular disease | 57 (27%) |
|  | Positive endocrine disease | 14 (6.7%) |
|  | Positive cardiovascular disease and endocrine disease | 48 (23%) |
| Tremor status | Negative | 129 (62%) |
|  | Positive | 80 (38%) |
| alpha-synuclein seeding amplification assay status | Negative | 18 (8.6%) |
|  | Positive | 191 (91%) |

BMI: body mass index
