# Supplementary material for: One patient, one destiny: A cluster analysis of the Parkinson’s progression Markers Initiative (PPMI) cohort
Source: Clin Park Relat Disord. 2026 Mar 21;14:100437. doi: 10.1016/j.prdoa.2026.100437 (PMC13049996; doi:10.1016/j.prdoa.2026.100437)
Supplement: Supplementary Data 3 [file mmc3.docx]

**Supplementaty Table 2. Patients’ characteristics after five years follow-up (Output variables)**

| Variables | | N = 209 |
| --- | --- | --- |
| Hallucination | Negative | 181 (87%) |
|  | Positive | 28 (13%) |
| Freezing of gait | Negative | 186 (89%) |
|  | Positive | 23 (11%) |
| Speech impairment | Negative | 207 (99%) |
|  | Positive | 2 (1.0%) |
| Arising from chair | Normal | 208 (99.5%) |
|  | Abnormal | 1 (0.5%) |
| Gait abnormality | Normal | 166 (79%) |
|  | Mild | 36 (17%) |
|  | Severe | 7 (3.3%) |
| Postural stability | Normal | 202 (97%) |
|  | Abnormal | 7 (3.3%) |
| Posture | Normal | 154 (74%) |
|  | Mild | 40 (19%) |
|  | Severe | 15 (7.2%) |
| Dyskinesia | Negative | 140 (67%) |
|  | Positive | 69 (33%) |
| Fluctuation | Negative | 109 (52%) |
|  | Positive | 100 (48%) |
| Dystonia | Negative | 179 (86%) |
|  | Positive | 30 (14%) |
| Cognitive impairment | Negative | 125 (60%) |
|  | Positive | 84 (40%) |
| Depression | Negative | 153 (73%) |
|  | Positive | 56 (27%) |
| Anxiety | Negative | 119 (57%) |
|  | Positive | 90 (43%) |
| Apathy | Negative | 160 (77%) |
|  | Positive | 49 (23%) |
| Dopamine dysregulation | Negative | 190 (91%) |
|  | Positive | 19 (9.1%) |
| Lightheadedness | Negative | 131 (63%) |
|  | Positive | 78 (37%) |
| Fatigue | Negative | 74 (35%) |
|  | Positive | 135 (65%) |
| Levodopa equivalent daily dose progression | Negative | 14 (6.7%) |
|  | Positive | 195 (93%) |
