# Supplementary material for: One patient, one destiny: A cluster analysis of the Parkinson’s progression Markers Initiative (PPMI) cohort
Source: Clin Park Relat Disord. 2026 Mar 21;14:100437. doi: 10.1016/j.prdoa.2026.100437 (PMC13049996; doi:10.1016/j.prdoa.2026.100437)
Supplement: Supplementary Data 4 [file mmc4.docx]

**Supplementary Table 3. General characteristics of study population across clusters**

| Variables | | **Cluster 1**  **(**N = 73) | **Cluster 2**  **(**N = 77) | **Cluster 3**  **(**N = 59) | **P-value** |
| --- | --- | --- | --- | --- | --- |
| Age (years) | | 59 [52.30–64.50] | 60.3 [55.4–66.6] | 62.9 [53.9–68.4] | 0.088 |
| Age category | <50 years | 15 (21%) | 8 (10%) | 6 (10%) | 0.134 |
|  | 50-65 years | 41 (56%) | 45 (58%) | 29 (49%) |  |
|  | 65-80 years | 16 (22%) | 24 (31%) | 24 (41%) |  |
|  | >80 years | 1 (1.4%) | 0 (0%) | 0 (0%) |  |
| Sex | Female | 32 (44%) | 29 (38%) | 17 (29%) | 0.207 |
|  | Male | 41 (56%) | 48 (62%) | 42 (71%) |  |
| Race | White | 67 (92%) | 66 (86%) | 50 (85%) | 0.776 |
|  | Black | 0 (0%) | 2 (2.6%) | 2 (3.4%) |  |
|  | Asian | 5 (6.8%) | 7 (9.1%) | 6 (10%) |  |
|  | Others | 1 (1.4%) | 2 (2.6%) | 1 (1.7%) |  |
| BMI (kg/m^2^) | | 24.79 [23.07–27.12] | 26.39 [23.6–29.56] | 27.87 [25.47–30.82] | <0.001 |
| BMI category | <18.5 kg/m^2^ | 1 (1.4%) | 0 (0%) | 0 (0%) | 0.001 |
|  | 18.5-25 kg/m^2^ | 39 (53%) | 28 (36%) | 13 (22%) |  |
|  | 25-30 kg/m^2^ | 29 (40%) | 31 (40%) | 29 (49%) |  |
|  | >30 kg/m^2^ | 4 (5.5%) | 18 (23%) | 17 (29%) |  |
| Genetic status | Negative | 43 (59%) | 54 (70%) | 42 (71%) | 0.231 |
|  | Positive | 30 (41%) | 23 (30%) | 17 (29%) |  |
| Comorbidities | Negative | 37 (51%) | 30 (39%) | 23 (39%) | 0.261 |
|  | Positive cardiovascular disease | 22 (30%) | 22 (29%) | 13 (22%) |  |
|  | Positive endocrine disease | 4 (5.5%) | 6 (7.8%) | 4 (6.8%) |  |
|  | Positive cardiovascular disease and endocrine disease | 10 (14%) | 19 (25%) | 19 (32%) |  |
| Tremor status | Negative | 44 (60%) | 48 (62%) | 37 (63%) | 0.950 |
|  | Positive | 29 (40%) | 29 (38%) | 22 (37%) |  |
| alpha-synuclein seeding amplification assay status | Negative | 7 (9.6%) | 6 (7.8%) | 5 (8.5%) | 0.925 |
|  | Positive | 66 (90%) | 71 (92%) | 54 (92%) |  |

BMI: body mass index
