# Supplementary material for: One patient, one destiny: A cluster analysis of the Parkinson’s progression Markers Initiative (PPMI) cohort
Source: Clin Park Relat Disord. 2026 Mar 21;14:100437. doi: 10.1016/j.prdoa.2026.100437 (PMC13049996; doi:10.1016/j.prdoa.2026.100437)
Supplement: Supplementary Data 5 [file mmc5.docx]

**Supplementary Table 4. Distribution of patients by genetic/alpha-synuclein status categories and progression trajectory clusters**

| Genetic/SAA group | Longitudinal trajectory clusters based on UPDRS III score progression | | | |
| --- | --- | --- | --- | --- |
|  | Slow | Intermediate | Rapid | Total |
| Negative genetic status, negative SAA | 0 | 2 | 0 | 2 |
| Negative genetic status, positive SAA | 44 (32%) | 53 (39%) | 40 (29%) | 137 |
| Positive genetic status, negative SAA | 9 (47%) | 3 (16%) | 4 (21%) | 16 |
| Positive genetic status, positive SAA | 20 (37%) | 19 (35%) | 15 (28%) | 54 |

SAA: Alpha-synuclein seeding amplification assay.

p = 0.358, Fisher’s Exact Test.
