# Supplementary material for: One patient, one destiny: A cluster analysis of the Parkinson’s progression Markers Initiative (PPMI) cohort
Source: Clin Park Relat Disord. 2026 Mar 21;14:100437. doi: 10.1016/j.prdoa.2026.100437 (PMC13049996; doi:10.1016/j.prdoa.2026.100437)
Supplement: Supplementary Data 6 [file mmc6.docx]

# Supplementary Table 5. Linear regression results (UPDRS III Δ as outcome)

| Model | Variable | Beta | 95% CI | p |
| --- | --- | --- | --- | --- |
| Univariate | BMI | 0.588 | 0.211 to 0.965 | 0.003 |
| Multivariable | Age at enrollment | -0.043 | -0.240 to 0.153 | 0.667 |
|  | BMI | 0.697 | 0.287 to 1.108 | 0.001 |
|  | Race | -0.476 | -3.040 to 2.088 | 0.716 |
|  | Sex | -1.669 | -5.304 to 1.965 | 0.369 |
|  | Comorbidities | -0.162 | -1.688 to 1.365 | 0.836 |
|  | Genetic | 0.807 | -3.142 to 4.756 | 0.689 |
|  | Tremor status | -0.013 | -3.609 to 3.584 | 0.994 |
|  | SAA status | 4.426 | -2.458 to 11.309 | 0.209 |

BMI: Body mass index; SAA : Alpha-synuclein seeding amplification assay.
